# Treatment of eating disorders in older people: a systematic review

**DOI:** 10.1186/s13643-021-01823-1

**Published:** 2021-10-25

**Authors:** Megha Mulchandani, Namrata Shetty, Agatha Conrad, Petra Muir, Beth Mah

**Affiliations:** 1 Hunter New England Mental Health Service, PO Box 833, Newcastle, NSW 2300 Australia; 2 Older People’s Mental Health Service, Hunter New England Mental Health Service, PO Box 833, Newcastle, NSW 2300 Australia; 3 Centre for Brain and Mental Health Research (CBMHR), The University of Newcastle, Callaghan, NSW 2308 Australia; 4 Karitane Residential Service, 138 The Horsely Drive, Carramar, NSW 2163 Australia

**Keywords:** Eating disorders, Older people, Treatment

## Abstract

**Background:**

Historically, eating disorders were not identified in older populations and it is only in more recent times that there is greater recognition of the existence of eating disorders among the elderly. This is despite the high level of morbidity and mortality associated with these disorders. Current guidelines focus on treatment of eating disorders within the adolescent and general adult age groups, without apparent concessions made for the older age group. The aim of this study was to review existing literature on the demographics and treatment of eating disorders in older people.

**Methods/design:**

A systematic review of the literature was conducted using CINAHL, MEDLINE, EMBASE, PsycInfo, Scopus, and Web of Science to identify publications focusing on treatment of eating disorders in people over the age of 65 years, age of diagnosis, gender distribution, treatment setting, and treatment outcomes.

**Results:**

A total of 35 articles (reporting on 39 cases) were relevant to our study, with 33 of the 35 articles being either case studies or case series. The mean age of participants was 73.2 years (range 66–94 years) with the majority (84.6%) being female. Most cases (84.6%) were diagnosed with anorexia nervosa, and 56.4% of all cases were reported as late onset (i.e., after age 40 years). The vast majority (94.8%) received treatment, of which 51.5% was hospital-based treatment. In case descriptions where improvement was reported, the majority described a multidimensional approach that included a combination of hospital admission, therapy and pharmacotherapy. Overall, 79.5% of cases who underwent treatment for an eating disorder improved, while 20.5% relapsed or died as a result of the complications from their eating disorder. There were significant inconsistencies and omissions in the way cases were described, thereby impacting on the interpretation of the results and potential conclusions.

**Conclusions:**

The information available on the treatment of eating disorders in people over the age of 65 years is limited. The quality of case reports to date makes it difficult to suggest specific assessment or treatment guidelines for this population.

## Background

Eating disorders represent a group of complex and chronic medical illnesses, with both psychological as well as medical features. They are characterized by abnormal eating behaviours that lead to significant morbidity and mortality [1]. They include anorexia nervosa, bulimia nervosa, binge eating disorder, and restrictive/avoidant food intake disorder, as well as other specified feeding and eating disorders as per the Diagnostic and Statistical Manual of Mental Disorders 5 (DSM 5) [2].

Eating disorders have long been thought to be illnesses that primarily affect young people [3]. Age has played a pivotal role in the diagnosis of eating disorders, in particular, anorexia nervosa [4, 5]. Since its initial description by Sir Gull [6], anorexia nervosa was primarily thought to be seen in people aged between 16 and 25 years. Even up until the 1980s, it was widely accepted that eating disorders were only seen in younger age groups, which was confirmed by DSM III, wherein the diagnosis of anorexia nervosa could only be made in people aged under 30 years [2]. Given this definition decades ago, it is possible that eating disorders have not been adequately recognised in the older age group.

There is no doubt that eating disorders have one of the highest morbidity and mortality rates amongst all mental illnesses, with anorexia nervosa having the highest mortality rate of all psychiatric disorders [7]. One meta-analysis estimated the mortality rates (deaths per 1000 members of the population per year) for anorexia nervosa, bulimia nervosa, and eating disorders not otherwise specified were at 5.1, 1.7, and 3.3, respectively [8]. In this study, one in five of those who died with anorexia nervosa did so through suicide, identifying psychological burden as a significant contributing factor relating to mortality [8]. Other research has indicated that anorexia nervosa’s crude mortality rate has increased from 5–10% to 17% over a 20-year follow-up [9].

With every edition of the DSM, there have been significant changes, reflecting changing views of the eating disorders over the decades. This is seen in changes from DSM III categorising eating disorders as those occurring in infancy, childhood, and adolescence to DSM IV, where eating disorders received their own category. It was in DSM IV that eating disorders were divided into anorexia nervosa, bulimia nervosa, and eating disorder not otherwise specified. It is of note that in DSM IV, having amenorrhoea was a necessary criterion for post-menarche women to meet a diagnosis of anorexia nervosa, thereby discounting the diagnosis of this illness in menopausal and post-menopausal women. This criterion has been removed with DSM V; in addition, binge eating disorder is now recognised as a separate diagnosis.

Historically, the elderly displaying eating disordered behaviours would have received a diagnosis of eating disorder not otherwise specified, a category reserved for those who do not meet full criteria for any specific eating disorder. Hence, an elderly woman with abnormal eating habits, a desire to lose or control weight, and a low score on body mass index would have been diagnosed with eating disorder not otherwise specified rather than anorexia nervosa using previous versions of DSM [1]. There has been limited research on eating disorder not otherwise specified—in spite of this being the most commonly diagnosed eating disorder subtype in adult populations [10]. This is likely to have contributed to further problems in estimating the prevalence of eating disorders in the elderly within the community. In the older population, it would be possible to misdiagnose eating disorders as a medical issue or a consequence of an organic problem. Another complicating factor in identification could be that adults are more adept at masking their illness [11] than their younger counterparts [1].

The awareness of eating disorders has increased in recent decades, with increased recognition of eating disorders amongst young “baby boomers” (born 1946–1964) [12], leading to an increase in the number of older adults presenting with eating disorders [1]. In addition, given that only a minority of individuals meeting the stringent criteria for eating disorders are seen in mental health care [3], it is plausible that many would not receive adequate treatment and continue to experience problems related to eating disorders as they age.

Recent studies show that 1.8–3.8% of community dwelling women over the age of 60 have indications of an eating disorder [13, 14], suggesting that the risk of these disorders are life-long concerns. With the limited evidence available, what is known about eating disorders in the elderly population is that anorexia nervosa is the most common form, 60% of people have additional comorbid psychiatric conditions, and there is a very high mortality rate of 21% [1]. While recognition of anorexia nervosa’s presence in the elderly population has increased, at present, there is limited understanding of its aetiology and clinical presentation and even less about treatment options.

The outcome for anorexia nervosa in adolescents is thought to be better than in adults, with recovery rates and chronicity of illness more favourable in studies with younger patients, and with a lower mortality rate [9]. The high risk of mortality and morbidity associated with eating disorder in younger age groups is well recognised. One could extrapolate, given limited physical reserve and poor ability to tolerate the sequelae of untreated or delayed treatment of eating disorders, that older people would be at increased risk of morbidity and mortality when compared with their younger counterparts. Hewitt et al. [15] examined over 10 million deaths in the USA, where anorexia nervosa was the primary cause of death or a secondary condition present at the time of death. They found that most deaths due to anorexia nervosa were in the elderly—10% in the 55–64 age group, 12% in the 65–74 age group, and 28% in the 85 and older group. This suggests that not only can eating disorders exist in the older age group, but that the risk of mortality and morbidity in this age group is significantly higher. Therefore, the timely recognition of eating disorders among older age groups appears to be of great importance [11].

Due to its well-recognised presence amongst adolescents and young adults, there have been well established guidelines for treatment of eating disorders in these age groups. These include specialist supportive clinical management (SSCM) [16], and The Maudsley model of anorexia nervosa treatment for adults (MANTRA) for adults over the age of 18 years [17]. Both approaches have been shown to be equally effective but with low recovery rates [18]. There are no equivalent guidelines addressing the treatment of individuals with eating disorders within the elderly.

Despite growing evidence for the presence of eating disorders in the older age group, as well as the recognition of greater risk of mortality and morbidity compared with the younger population, little is known about effective treatment strategies, with no current guidelines available to assist clinicians. Given the comparative variety of complexities seen in the elderly versus young adults and middle-aged individuals, this systematic review aims to identify, synthesize, and critically evaluate the scientific literature on eating disorders in elderly populations, in order to improve the understanding of treatment in the elderly.

## Methods

The methodology to perform this systematic review was developed according to recommendations from the Preferred Reporting Items for Systematic Reviews and Meta Analyses (PRISMA) guidelines. The study was registered with PROSPERO (www.crd.york.ac.uk/PROSPERO) with the registration number of CRD42016042361 on 07/07/2016 and with title of ‘Eating disorders in the elderly’.

### Search strategy

There were two steps used in the search strategy. Firstly, the Cochrane Database of Systematic Reviews, The International Register for Systematic Reviews, PROSPERO, and Joanna Briggs Institute were searched to identify any recent or past systematic reviews meeting the selection criteria (i.e., investigating the treatment of eating disorders in older people). Articles were included in the review using the following terms: ‘Eating disorder’, ‘Bulimia Nervosa’, ‘Anorexia Nervosa’, and ‘Binge Eating Disorder’. This process allowed for inclusion of individual articles from systematic reviews, as well as any systematic review as an initial step of the review process.

The second step involved searching seven major databases including: CINAHL, MEDLINE, EMBASE, PsycInfo, PubMed, Scopus, and Web of Science from the initial date of each database through to July 2019. The following terms were used: ‘Eating disorder’, ‘Anorexia Nervosa’, ‘Bulimia Nervosa’, and ‘Binge Eating Disorder’, with studies limited to Human, English language, and age greater or equal to 65 years old. Due to age not being an option both in Scopus and Web of Science, the following terms were used: ‘Eating disorder and old age’, ‘Eating disorder and elderly’, ‘Eating disorder and geriatric’, ‘Anorexia Nervosa and old age’, ‘Anorexia Nervosa and elderly’, ‘Anorexia Nervosa and geriatric’, ‘Bulimia Nervosa and old age’, ‘Bulimia Nervosa and elderly’, ‘Bulimia Nervosa and geriatric’, ‘Binge Eating Disorder and old age’, ‘Binge Eating Disorder and elderly’, and ‘Binge Eating Disorder and geriatric’. The search was conducted by the primary reviewer.

### Inclusion criteria

Articles were included if the study (a) had an eligible study design, including systematic reviews, randomised controlled trials, non-randomised controlled trials, quasi-experimental design, cohort (observational) studies, case reports, case series, and case controlled studies; (b) explored the treatment of eating disorders; (c) had patients/participants aged 65 years or greater; and (d) had a diagnosis of an eating disorder.

### Exclusion criteria

Articles were excluded if (a) they were in other languages than English or (b) studies involved animals or laboratory observations.

### Selection of studies

Search results were downloaded into an EndNote database. The primary reviewer checked the titles and abstracts of all obtained studies ensuring that the articles included contained the correct search terms and looking at the inclusion criteria which included the diagnosis search terms. Two reviewers independently checked titles and abstracts of all obtained studies, retaining studies meeting the inclusion criteria. This was then cross-referenced by the two independent reviewers and any disagreements were then discussed with a 3rd independent arbiter. Studies that were included by title and abstract were further reviewed in full text, again independently by the 2 reviewers. Any disagreements were again cross-referenced and discussed with a 3rd independent arbiter as necessary.

### Assessment of quality

It was not possible to assess the risk of bias, as all of the studies included were case studies. However, the Critical Appraisal Checklist for case series and case reports [19] was used in deciding whether to include or exclude the studies in this review. In addition, the quality of the case reports/series studies was evaluated by two independent reviewers based on a combination of clinical judgement and whether sufficient detailed information was included (for example age, gender, diagnosis, initial weight, treatment, whether the person improved by weight gain, and how much of weight they gained), allowing for studies to be classified as either “good” or “poor”. Studies were classified as “good” if they had a clear description of the illness, including the initial weight prior to treatment and after treatment, what treatment the patient received, and the outcome in relation to weight and discharge from hospital. Studies that were classified as “poor” typically had missing outcome information in relation to weight gain/loss and whether the patients were discharged from hospital.

### Data extraction

Two reviewers independently extracted information about studies from the Endnote database. The following data were extracted:

1. Publication details: authors and year of publication;
2. Study design: type of study (randomised controlled trials, non-randomised controlled trials, quasi-experimental design, cohort (observational) studies, case reports, case series, and case controlled studies, recruitment methods, and data collection methods;
3. Participants: number of participants, and participant demographic characteristics (including age greater or equal to 65 years and gender);
4. Diagnosis information: diagnosis (including DSM V eating disorder diagnoses);
5. Clinical features (weight, body mass index, dietary restrictions, and medical comorbidities and complications); and
6. Treatment (including medications, psychological, inpatient and community treatment)

### Outcome

The outcomes of this review will firstly identify any associations between eating disorder type and treatment modality and examine any weight gain due to potential interventions and mortality rates within the elder population diagnosed with an eating disorder. In addition, it will also examine the frequency of comorbid psychiatric conditions among the elderly diagnosed with an eating disorder.

### Data synthesis

**Fig. 1:**
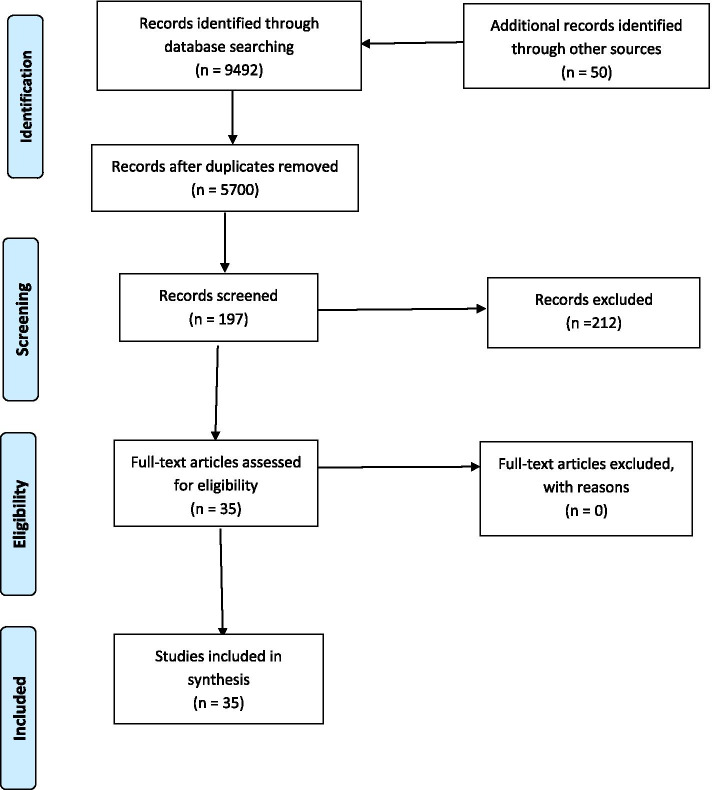
PRISMA 2009 flow diagram [19]

As there were no RCT or quasi-experimental observational studies available in this area, a meta-analysis was not conducted, nor was it possible to examine measures of treatment effects, as all the studies reviewed were case studies. Data extracted from eligible studies was synthesised using a narrative approach. The synthesis aimed to describe the existing body of literature, identifying any strengths and gaps in the diagnosis and treatment of eating disorders among the elderly. Study selection strategy and the results of the literature search are depicted in Fig. 1. Extracted relevant data for each study is summarised in Table 1. The findings are further summarised below in a narrative interpretation focused on the primary aim of this review, to provide a better understanding of eating disorders in elderly populations in order to improve our undertaking of diagnosis and treatment in this population.

**Table 1:** Characteristics of included studies

Study details (author, year) Study type [ref] Rated quality	Age (gender)	Diagnosis^**a**^	Clinical features^**a**^	Treatment^**a**^	Outcome/mortality	Conclusions ^**b**^
(Bernstein, 1972) Case study [20] GQ	94 (F)	AN	*Weight 36 kg *Refusal to eat *Symptoms: uncooperative/agitated, depressed, paranoid	*ECT, intravenous feedings	*Weight gain of 2.7 kg, improve oral intake, no paranoid thinking	
(Launer, 1978) Case report [21] GQ	70 (F)	AN (BP)	*BMI 10.3 *Fear of gaining weight despite being thin, *Caloric restriction, laxative abuse	*Medical inpatient admission *Chlorpromazine and clomipramine (for obsessional thoughts) *Structured behavioural regime observed during mealtimes, confined to bed and rewards (mobility) made contingent on an increase in weight	*Weight gain of 14 kg & more amenable. *Weight loss with less restrictive care. *Remained in hospital (6-month admission)	*AN can exist in the elderly, without a past diagnosis of AN
(Price, 1985) Case study [22] GQ	68 (F)	AN (PT)	*BMI 17.9 *Weight loss of 9.5 kg *Body image disturbance *Food restriction, laxative abuse, *Mild nutritional anaemia	*Behavioural plan which provides positive reinforcers for gaining weight & negative for either losing or failing to gain weight.	*Weight gain of 9.5 kg over the following 10-12 months *Loss to follow up after moving away	
(Ronch, 1985) Case study [23] GQ	75 (M)	AN	*BMI 13.7 *Distorted body image *Food restriction, self-induced vomiting, laxative abuse, secretive food disposing *No physiological abnormalities	*Admission to intermediate care facility *Dietician review but declined nutritional supplements. *Psychological therapy included CBT *Weight goals with medical transfer if falls below threshold weight. *Food and nutrition plan devised in conjunction with patient. Close supervision of food, nutritional supplement intake & food consumption in dining room with others. *Tranxene 3.75 mg every 2nd night for anxiety	*Weight gain of 5.5 kg & ceased disposing of food	*AN can survive and become a predominant feature of late-life adaptation patterns. *Treating AN in older population can be modestly successful with a coordinated interdisciplinary, institution-wide effort. *Putting AN into life context helps to better understand its aetiology and lifelong role as an adaptive attempt in the face of narcissistic injury, problems with control, anger and self-esteem, &possibly as a way to try to cope with some of the stresses of ageing.
(Barry, 1987) Clinical comment [24] PQ	78 (M)	IB	*Binging and purging	*CBT	*Cessation of purging, but relapse after termination of individual psychotherapy. *Social skills training	*Feasibility of psychologically based eating disturbances among the institutionalised aged. Proper diagnosis is essential for both targeting appropriate referrals and for effective treatment planning.
(Ramell, 1988) Case study [25] GQ	67 (F)	AN,	* BMI: 18.2 *Fear of gaining weight *Distorted body image *Caloric restriction *Purging	*Admitted to medical ward *Small doses of Chlorpromazine & insulin, *Free choice of diet *Daily monitoring *Laxative discouraged and replaced with stool bulking agents	*Rapid weight gain of 7 kg and stable at 6 months but preoccupied with caloric counting	*AN can develop in patients of any age
(Fenley, 1990) Case study [26] GQ	75 (F)	AN	* BMI 20.4 *Weight loss *Fear of being overweight * Body image disturbance *Chronic laxative abuse *Numerous serious medical complications from ED	*Medical stabilisation *Inpatient psychiatric admission *Behavioural management, individual psychotherapy, family therapy, group therapy, therapeutic milieu *Escalating oral *Alprazolam, poor tolerance of nortriptyline *After discharge, outpatient psychiatrist and physician follow-up	*Weight gain of 2 to 36 kg & maintenance of weight.	
(White, 1990) Case report and literature review [27] GQ	82 (F)	AN	*BMI 11.7 *Body image disturbance *Restriction of food intake, purging	*Medical admission *Started on isocarboxazid 15 mg daily	*Gained 6-kg weight with improvement in oral intake. *Discharged from hospital & doing well with out-patient supervision *Two months after discharge, died from pneumonia	*There is lack of guidance in literature as to the best treatment and anticipated outcome of AN in old age *Pharmacological treatment of AN has had limited success. *Antidepressants should be considered, starting at very low doses and slowly increased. *No epidemiological data about prognosis of AN in elderly exists.
(Cosford,1991) Letter to editor [28] PQ	73 (F)	AN	*Weight loss, *Fear of weight gain *Body image disturbance	*Inpatient psychiatry admission with strict dietary regime, outpatient follow-up	*Weight restoration and maintenance of weight	
(Nottingham,1991) Case study [29] GQ	66 (F)	AN (PT)	*BMI 11.9 *Body image disturbance *Purging and laxative abuse *Medical complications from AN	*Inpatient admission *Doxepin *Hyperalimentation	*Discharged after weight gain of 7 kg, but relapse with weight loss *Chronically remained unwell with AN and then death due to complications from AN	*Need to be aware of AN in elderly and that coordination and education is needed to provide adequate treatment.
(Riemann, 1993) Case study [30] GQ	72 (M)	AN (PT)	*BMI 16.5, weight loss of 31 kg *Fear of becoming fat *Low body weight *Body image disturbance *Purging, laxative abuse, excessive exercise, food restriction	*Declined inpatient admission & did not engage in community follow-up		
(Wiederman,1995) Clinical comment [31] PQ	86 (F)		*Weight 57 kg, weight loss of 18 kg *Food restriction *No physiological abnormality identified	*Dietician and physician involvement *Soft food initially and then gradual introduction of normal diet *Reinforcement of recovery *Involvement in nursing home social support group	*Not known	*To consider eating disturbances in elderly from a mental health perspective
(Beck, 1996) Case series [32] GQ	77 (F)	AN(RS)	* BMI 13.5 *Weight loss of 19 kg *Body image disturbance *No physiological abnormalities	*High caloric supplement diet, *Monthly psychiatry follow-up *Food diary	*Weight stabilised at 41–43 kg, *Working as volunteer	
(Beck, 1996) Case series [32] GQ	80 (F)	AN (BS)	* BMI 21.0 *Weight loss 27 kg *Refusal to maintain body weight *Food restriction *Binge behaviour with compensatory fasting	*Declined treatment		
(Pobee, 1996) Case study [33] GQ	82 (F)	AN(PT)	*BMI 15.5 *Weight loss of 13 kg *Body image disturbance *Food restriction, *Purging and laxative	*Prescribed oral supplements as per dietician but refused and continued to lose weight *Failed trial of Ritalin *Conservative management with goal to increase weight. *Encouragement by staff at meal times	*“Remained a poor eater and continued to be very critical of food”	
(Hsu,1988) Case 1 [34] GQ	72 (F)	AN	*BMI 12.6 *Weight loss of 17.7 kg *Fear of gaining weight *Body image disturbance	*Admitted to psychogeriatric ward *Behaviour modification but difficult to engage	*Weight gain of 4.5 kg, but relapsed soon after discharge and admitted to another teaching hospital	*Outcome of ED in elderly just as varied as younger patients. AN seems to do badly whereas people with BN seem to do better with treatment *ADTs are worth the trial even in absence of concurrent depression. If one ADT is ineffective, trial of second or third should be made. *Treatment should be directed at all major areas of dysfunction, since improvement in one symptoms is not necessarily related to that of another
(Hsu,1988) Case 2 [34] GQ	67 (F)	BN (PT)	* BMI 18.7 *Weight loss of 5.5 kg *Trying to control weight *Fear of weight gain *Denial of illness *Binging and vomiting, laxative abuse	*CBT *Continuation of medications (trazadone, diazepam, dextroamphetamine) *Tranylcypromine dramatically improved dysthymic symptoms	*Improvement of binging and vomiting, unknown weight gain	*Outcome of ED in elderly just as varied as younger patients. AN seems to do badly whereas people with BN seem to do better with treatment *ADTs are worth the trial even in absence of concurrent depression. If one ADT is ineffective, trial of second or third should be made. *Treatment should be directed at all major areas of dysfunction, since improvement in one symptoms is not necessarily related to that of another
(Nagaratanam, 1988) Case study [35] GQ	70 (M)	AN	*Low body weight, 31 kg *Food restriction, *Medical complications from AN	*Nasogastric feeding	*Weight gain of 4.2 kg over 15 days, but then died suddenly of unknown cause	
(Nicholson, 1998) Literature review with case study [36] PQ	76 (F)	AN	*Weight 36 kg *Intense fear of gaining weight *Body image disturbance *Medical complications from AN.	*Psychiatric inpatient admission Psychoeducation *Small regular attractive meals and extra feeds	*Deterioration in physical state leading to medical admission. *Subsequently discharged to nursing home where she eventually died	*Treatment modalities similar to those of younger people: pharmacological and psychological, given general frailty of older people, best to admit to hospital for treatment of ED. *Around 2/3 of patients respond to treatment, which in line with younger people.
(Russell, 1998) Case study [37] PQ	69 (F)	AN (PT)	*BMI 11.3 *Weight loss of 17.5 kg *Fear of gaining weight *Food restriction and purging *Medical complications	*Inpatient medical admission *Nasogastric refeeding with supplements	*Weight gain to 39 kg, however died from complication related to metastatic lung carcinoma	*AN in postmenopausal patients is an unusual condition and be regarded cautiously as a diagnosis of exclusion.
(Wills , 1998) Case series [38] GQ	74 (F)	AN	*BMI 12.7 *Fear of gaining weight, *Body image disturbance *Restriction of food intake *No physiological abnormalities identified	*Managed in community-based old age psychiatry with “a programme”	*Weight gain of 7.8 kg. *Died aged 82 in nursing home	*AN is an attempt to take control when other aspects are out of control *Age should no longer appear as a diagnostic criteria for AN
(Wills Case 2, 1998) Case series [38] GQ	67 (F)	AN	*BMI 15.6 *Body image disturbance *Food faddiness	*Group psychotherapy *Community psychiatric follow-up	*Followed up for 7 years. *Weight remained below 40 kg but able to return to normal lifestyle	*AN is an attempt to take control when other aspects are out of control *Age should no longer appear as a diagnostic criteria for AN
(Wills Case 6, 1998) Case series [38] GQ	75 (F)	AN	*BMI 13.7 *Preoccupation with food, laxative abuse	*3 hospital admissions in 6 years *Non-compliant	*Lives with husband	*AN is an attempt to take control when other aspects are out of control *Age should no longer appear as a diagnostic criteria for AN
(Hill, 2001) Case study [39] GQ	77 (F)	AN	*BMI 16.5 *Weight loss of 21 kg *Restrictive food intake and exercise *Other symptoms: depressive symptoms *No physiological abnormalities	*Inpatient psychiatric admission *ECT (to treat depressive symptoms)	*Weight gain of 2 kg, eating regular and full meals *Euthymic mood	
(Mermelstein, 2001) Case study [40] PQ	92 (F)	AN	* BMI 14.7 *Body image disturbance *Restrictive intake, laxative abuse, excessive exercise *Medical complications from AN	*Inpatient admission *Treatment based on for younger anorectic patients. *Cognitive therapy focusing on body image, food- and weight-related constructs. *Privileges based on weight gain *Paroxetine 20 mg to lessen obsessional attention to eating *Dietician review and caloric supplementation	*After 3 months, gained enough weight to be discharged to a minimal assisted living facility. *Doing well 6 months post discharge	*The need to heighten diagnostic sensitivity for AN and other ED at any age
(Parke, 2008) Case study [41] GQ	72 (M)	EDNOS	*BMI 16.7 *Disturbed body image *Denial of seriousness of illness. *Dietary restriction, preoccupied with food, excessive exercise to control weight *Medical complications	*Inpatient psychiatric admission *Multivitamin, iron, and mineral therapy. *Fluoxetine *Weighed regularly and monitored closely after meals. *Physical activity restricted	*Gain of 10 kg *No improvement in cognition. *Discharged to nursing home	* Need for diagnostic awareness regarding ED in patients of all ages and both genders
(Lapid, 2010) Review with case study [1] PQ	73 (F)	AN	*Low body weight *Preoccupation with weight *Laxative abuse	*Inpatient psychogeriatric admission *ECT *Continuation of antidepressant *Behavioural intervention *Psychoeducation *Calorie counting *Meal observations and 1 h post prandial	*Weight gain, improved appetite, and caloric intake.	*ED occur in elderly, with AN being the most common. *Depression is most common comorbid psychiatric condition *Combination of pharmacological and behavioural interventions may be successful.
(Cwikel, 2011) Case study [42] GQ	67 (F)	AN	* Weight: 59 kg *Concerns about body image *No physiological abnormalities	*CBT and follow-up with nutritionist	*Currently stable. *Nil relapse. *Resumption of normal eating patterns	
(Lozano, 2011) Case study [43] PQ	79 (F)	AN	*BMI 12.7 *Food restriction *Purging *Other symptoms: low mood, delusions *Medical complications from AN	*Regular diet with monitoring of intake. *Initiation of fluoxetine and mirtazapine *Physical therapy *Occupational therapy	*Unknown	
(Main, 2011) Letter to editor [44] PQ	75 (F)	AN (RT)	*BMI 14.8 *Food restriction *No physiological abnormalities,	*Family therapy *Community dietician *Medical and psychiatric input from primary care and community psychiatry	Unknown	
(Lehman, 2012) Case study [45] PQ	77 (F)	AN	*BMI 16.4 *Refusal to maintain normal body weight *Fear of gaining weight *Denial of seriousness of illness *Laxative abuse *Other symptoms: mood symptoms	*Minimum weekly outpatient individual psychotherapy (including CBT and Knight’s Contextual, Cohort-Based, Maturity, Specific-Challenge (CCMSC) psychotherapy model) *2 days/week of partial hospitalization program *Clinician built relationship with all involved stakeholders *Maintenance treatment included asenapine, zolpidem, aripiprazole, mirtazapine, buproprion XR, lorazepam, benztropine. *Increase in dose of antidepressant.	*Eating 2 meals/day, increased functional independence, improved social engagement *Ongoing mood fluctuation (related to schizoaffective disorder)	
(Lwin, 2014) Case study [46] GQ	73 (F)	BN (PT)	*Weight loss of 11.3 kg *Binging and purging *Repetitive pacing, agitation *No physiological abnormalities	*Mirtazapine 30 mg started and within a week, self-induced vomiting and pacing abated. *Patient gained weight	*Bulimic symptoms were in remission 1 year follow-up	
(Malik, 2014) Case study and literature review [47] GQ	81 (M)	AN(PT)	*BMI 17 *Weight loss of 12 kg *Preoccupied with weight *Purging *Medical complications (hypophosphataemia, hypoalbuminaemic, hypoalbuminaemic), CT brain showed small vessel ischaemia in white matter	*Inpatient care *Advice from tertiary eating disorders service *Non-restrictive approach focusing upon dietary supplementation. *Follow-up with GP for monitoring of nutritional state.	*BMI improved to 17.5	*AN is an uncommon cause of unexplained weight loss in the elderly, but may be under-recognised and associated with a high level of mortality
(Taylor, 2015) Case study [48] PQ	80 (F)	AN	*BMI 11.5. *Weight loss of 8 kg *Restricted food intake *Medical complications from AN	*Dietician review and started on dietary supplementation and intravenous fluids *Started on an antidepressant (name not provided)	*Started to gain weight but then developed hospital-acquired pneumonia and died shortly afterwards	*AN in elderly remains underdiagnosed *ED should be a differential diagnosis of unexplained weight loss in the elderly *Symptoms of AN are the same as younger age group *Greater awareness of ED amongst healthcare workers is needed to prevent significant morbidity and mortality *There is need for service provision for this population group *Controlled trial investigation is needed to understand best treatment approach for older patient with an ED
(Aziz, 2017) Review [49] GQ	73 (F)	ED	*BMI 13.75 *Restriction of food intake	*Mirtazapine *Supportive care through SMHSOP *CMHT, day hospital *Dietetic support	*Recovered over a 6-month period to BMI of 18	*Treat both ED and comorbid psychiatric disorders *Pharmacological and behavioural interventions *Working on psychological problems (focus on psychotherapy) more effective than focussing on food choice and addressing weight loss *Supportive counselling *Psychoeducation on normal physical changes with ageing *Address physical health needs *Psychoeducation to families *Day programmes: dine with others, increase social interactions, physical health rehab. *Treat comorbid psychiatric disorders
(Aziz, 2017) Review [49] GQ	67 (F)	AN (AT)	*BMI 14 *Restriction of food intake *Other symptoms: felt full, not hungry, affect indifferent and lacked drive	*Psychotherapy *Support from local eating disorders services	*BMI reached 16.5	As above
(Aziz, 2017) Review [49] GQ	89 (F)	AN	*BMI 14 *Restriction of food intake	*ADT antidepressant medication	*Responded to this	as above
(Zayed, 2017) Report PQ [11]	66(F)	AN	*BMI 17.5 *Fear of gaining weight *Body image disturbance *Restriction of food intake *Denial of seriousness of current low body weight *Physical complications: oedema, ECG changes, pathology changes (low haematocrits, glucose, calcium, protein, albumin)	*Enteral feeding	*Developed refeeding syndrome, with multi-organ failure resulting in death	*Combination of both behavioural and pharmacological treatment found to be most successful.
(Hasan, 2017) Case report [50] PQ	76 (F)	AN	*Restriction of food intake *Fear of gaining weight *Body image disturbance	*CBT (with exposure and response modification): 8 weeks *Group programming (experiential therapy, nutrition, nursing) *Psychoeducation (body image & nutrition education) *Dietitian and meal plan *Managing physical complications *Venlafaxine 187.5 mg daily	*Gained 17 lbs *Increased caloric intake *Home health nurse to help with managing medications	*Support for the use of exposure-based CBT, coupled with behavioural activation, medical consultation
(Fahs, 2013) Book Chapter [51]	General comments made by author “An older adult generally presents with a greater severity of disordered eating but has fewer body image difficulties. Tend to deny symptoms. Use of over the counter meds, prescribed or illicit substances to produce weight loss, making difficult life transitions, inability to mourn major losses, fear of aging, feeling in competition with younger generations, setting unrealistic goals for oneself”.			*Before making an ED diagnosis, need to rule out other medical issues for symptoms and also medical consequences of ED. *Psychological interventions to understand how individual is coping with natural life transitions and one’s own mortality. *Nutritional counselling for meeting nutritional requirements *Stretching, aerobic, and strengthening exercises are recommended		
(Blake, 1996) Proceedings [52]				*“Medical intervention” required. *“A MDT approach, focusing on medical, psychological, and dietary issues, with antidepressants and ECT being useful in some cases.”		

^a^*ED* eating disorders, *AN* anorexia nervosa , *BN* bulimia nervosa, *BED* binge eating disorder, *EDNOS* eating disorder not otherwise specified, *BP* binge purging, *PT* purging type, *IB* idiosyncratic bulimia, *RS* restrictive subtype, *BS* bulimic subtype, *AT* atypical type, *ECT* electroconvulsive therapy, *BMI* body mass index, *CBT* cognitive behavioural therapy

^b^There were some studies without conclusions. Rated quality of case report/series: *GQ* good quality; *PQ* poor quality; (see text for criteria applied)

## Results

There were no systematic reviews exploring the treatment of eating disorders in older people identified in the international register for systematic reviews, PROSPERO, Cochrane Database of Systematic Reviews, or the Joanna Briggs Institute. There was no consistent agreement on the definition of what are “late onset” eating disorders within the literature. For our study, patients were identified as having an onset of eating disorders in later life (i.e., patients with eating disorder who were older than 65 years), in order to align with the national age standards for older people.

A total of 9492 records were identified through database searching, and after removal of duplicates, 5700 articles remained. All records were screened by two independent assessors, in order to include and exclude by title/abstract and then based on full text. After excluding articles by title/abstract, a total of 197 articles were identified through the databases. Following this, the reviews from databases were hand-searched for original articles, and 50 additional articles were included based on their title/abstract. Full articles were then sourced for the combined total of 247 references identified. The same two independent assessors separately read articles and identified those that met the inclusion criteria. A third independent assessor resolved any disagreements. At this stage, 212 studies were excluded, as the participants were < 65 years old or the study was not published in English. A total of 35 studies were identified as relevant for our systematic review (see Fig. 1).

Table 1 summarises the characteristics of the retained studies. Of the 35 studies that were included in our systematic review, 33 studies discussed cases, and the other two articles made only general comments about the treatment of older people with an eating disorder. Thus, all included studies were Level IV (case series) or less with respect to the National Health and Medical Research Council (NHMRC) guidelines for examining the level of research evidence [53]. In the two non-case descriptor studies [54, 55], both commented on the multifaceted approach to treatment of eating disorders in older people, which includes medical, psychological, and dietary management.

In total, 39 cases were described in the 35 selected studies, of which 69.2% (27 case reports) were classified as “good” quality, and 30.8% (12 case reports) were classified as poor quality based on our criteria. Among the 39 cases reported, 33 (84.6%) were females and 6 were males (15.4%). The age of patients at the time of assessment varied from 66 to 94 years with a mean age of 73.2 years. Female age range was 67–94 years, and male age range was 70–81 years. Of the 39 cases, 33 (84.6%) were diagnosed with anorexia nervosa, 3 with bulimia nervosa (7.7%), and 3 (7.7%) cases were given an unspecified eating disorder diagnosis. When considered by gender, of the 33 women, 29 (87.9%) were diagnosed with anorexia nervosa, 2 (6.1%) with bulimia nervosa, and 2 (6.1%) with an unspecified eating disorder diagnosis. As for the 6 male cases, 4 (66.7%) were diagnosed with anorexia nervosa, 1 (16.7%) with bulimia nervosa and 1 (16.7%) with an unspecified eating disorder.

The stated age of onset of eating disorder was not always made clear, but in 22 out of the 39 cases (56.4%), the onset of illness was reported to have occurred after the age of 40 years. Eighteen of the 33 cases (54.5%) with anorexia nervosa had an onset after the age of 40 years, whilst with bulimia nervosa, there were two out of the three (66.6) whose illness onset was after the age of 40 years, and 2 of 3 of unspecified eating disorder had a later onset of illness. Eight of the 39 cases died during follow-up (20.5%), but not all these deaths were reported to be a complication of the eating disorder. In 3 case studies, no cause of death was specified.

Of the 39 cases, 21 were reported to have comorbid or prior psychiatric illnesses recorded, with many cases having more than one comorbidity. Depression (or depressed mood) was reported as the most common past illness or comorbidity, seen in 12 of 21 cases (57.1%). Past history of eating disorder was reported in 6 cases. Anxiety was reported in 2 cases. Personality traits affecting the patient were reported in 2 cases (obsessional in one case and schizotypal in another). Dementia was reported in 2 cases. Schizophrenia/Schizoaffective disorder was reported in 2 cases.

Majority of the patients (37/39 cases, 94.8%) received some form of treatment. Of those who received treatment, 51.5% (17/33 cases) of patients with anorexia nervosa were admitted to the hospital, and two patients with the same diagnosis refused treatment. However, from the case descriptions, the location of admission and the type of treatment received was unclear at times; in these cases, it would be reasonable to assume that such treatment would generally only occur in an inpatient setting [35, 48]. None of the patients who were admitted to the hospital had a diagnosis of bulimia nervosa, whilst 33.3% of admitted patients (1/3) had a diagnosis of an unspecified eating disorder.

Out of the 33 cases of anorexia nervosa in Table 1, 22 (66.7%) cases reported weight gain as a result of treatment, with an average weight gain of 7.9 kg, whilst 6 (18.2%) cases reported no change in weight, and 5 (15.2%) cases reported no outcome. At follow-up, 25 (75.8%) cases were still alive and 8 (24.2%) cases were reported as either relapsed or died (either from complications of their eating disorder or due to an unrelated cause). All three cases of bulimia nervosa were reported to have improved, with one case having gained weight, but the amount of weight was not specified, with the other two cases reporting changes in symptoms but not a weight outcome, with one of these cases relapsing. All three cases with bulimia were alive in the follow-up period. Of the cases where the diagnosis was not specified, two were reported to have improved by gaining weight, with an average weight gain of 6.5 kg over the treatment period, whilst the outcome for the other case was not reported.

Because most of the cases discussed anorexia nervosa, we looked at the factors leading to improvement and factors that may prevent remission in these cases. Almost all of the cases of anorexia nervosa that showed an improvement had adopted a multidimensional approach to treatment, with one even taking on the adolescent treatment model [40]. Of the 16 cases in this group, 8 (50.0%) patients were admitted to hospital (either a medical or psychiatric unit).

In many of the cases of anorexia nervosa, the following psychological therapy modalities were identified: cognitive behavioural therapy (CBT) (most commonly used), individual psychotherapy, family therapy, supportive therapy, psychoeducation, and group therapy. Whilst some cases described adopting a reward system (privileges only with weight gain), others took on a non-restrictive approach to care. In many reported cases, the dietician was consulted, and food supplements were instituted, mostly orally, but in one case, intravenously [20]. Many case studies described treatment with medications that included mirtazapine, bupropion, paroxetine, chlorpromazine and insulin, alprazolam, lorazepam, clorazepate, and venlafaxine. In three patients, electroconvulsive treatment was administered [1, 20, 39], with reported improvement in symptoms; however, in these cases, there was also evidence of comorbid (or primary) depressive symptoms. Outpatient follow-up was commonly arranged for people who had been inpatients. Other approaches included keeping a food diary, partial hospital day programme and, in two cases, advice sought from a tertiary eating disorder service [47].

With regard to case reports of anorexia nervosa where patients improved, but then relapsed or died as a result of their illness (8 of 33 cases, 24.2%), factors that may have contributed to a lack of sustained improvement appear to have been chronicity of illness (between 5 to 47 years), very low body mass index (between 10.3 to 12.6), and poor engagement with a treatment plan. In anorexia nervosa patients who did not show any response to treatment, similar characteristics were also associated with a lack of response.

In the reported cases of bulimia nervosa, patients were treated with either CBT, medication alone, or a combination of CBT and medication (tranylcypromine or mirtazapine). The two case reports of bulimia nervosa [34, 46] which received antidepressants showed sustained improvement, whereas the patient receiving psychotherapy alone relapsed after treatment was ceased [24].

Overall, 79.5% (31/39) of the identified cases with an eating disorder were reported to have improved with treatment, while 20.5% (8/39) of cases either relapsed or died due to complications from their eating disorders. Comorbidity was a strong factor in many cases, in particular mood disorder [1, 20, 39, 45, 46], with the possibility that disordered eating was secondary to the mood disorder, rather than a primary eating disorder diagnosis, and thus treatment for mood disorder led to an improvement in eating habits.

We also examined the content of conclusions made by the authors of the reports and classified content into three broad categories. These categories included (a) a lack of awareness of the existence of eating disorder within the older age population, (b) the need for consideration of eating disorder as a differential diagnosis for unexplained weight loss in the elderly, and (c) continued need to be assertive in ruling out organic causes for unexplained weight loss, as medical causes for weight loss are common in the elderly.

## Discussion

Our study, a systematic review of the treatment of eating disorders in the elderly, is the first of its kind and has highlighted several issues. Firstly, there is little published information on the treatment of eating disorders in older people and all that is published is mostly case studies/series or clinical commentaries. Whilst it was not possible to assess the risk of bias, we were able to classify all of the studies as either “good” (69.2%) or “poor” (30.8%) quality based on a combination of clinical judgement and detailed information contained within the study, particularly around outcome achieved and treatment provided. Secondly, of what is known, there is little consistency in treatment approaches. Finally, prognosis for this disorder may be better than expected. There is no consistent agreement on “late onset” eating disorders within the literature; however, our study, identified later life eating disorders to be after the age of 65 years in order to align with the national age standards for older people.

This systematic review (including studies published 1972–2019), found 35 articles, with 39 case reports discussing the treatment of eating disorders in an older group. The impact of changes made to the Diagnostic System over a 40-year period, along with improved treatments for complex clinical presentations involving comorbidity (such as depression and personality disorders), undoubtedly have made a significant contribution to our clinical understanding, treatment approaches and clinical practice, which in turn may have led to improved prognosis. However, the small of number of available studies means that we need to be careful about interpreting the results obtained.

This study showed that 84.6% (33/39) of cases were diagnosed with anorexia nervosa, and 15.4% (6/39) of cases were diagnosed with either bulimia nervosa or eating disorder not otherwise specified. Our results were similar to those described by Lapid [1], who reported that 81% of eating disorders were diagnosed with anorexia nervosa. This differs from the prevalence seen universally in the general adult population, wherein binge eating disorder is most prevalent, followed by bulimia nervosa and finally anorexia nervosa [3]. The difference may be due to the fact that elderly cases were published when they came to the attention of health professionals, due to the extreme health complications [3]. Considering that anorexia nervosa is associated with the highest mortality and morbidity rates [9] of all psychiatric illnesses, it can be argued that anorexia nervosa is the most commonly recognised disorder compared with other eating disorders with fewer complications or more subtle clinical features.

The outcome for adolescents with anorexia nervosa is thought to be better than in adults, with recovery rates and chronicity of illness being more favourable in studies with younger patients, along with a lower mortality rate [9]. Research evidence shows that two thirds of young people with anorexia nervosa treated with a combination of pharmacological and psychological interventions have positive outcomes, with the rate of response to treatment being similar among older people ]32]. Other researchers agreed that the combination of pharmacological and behavioural management was the treatment of choice [1]. However, Hsu (1988) reported that older people with anorexia nervosa had a relatively worse outcome compared with people with bulimia nervosa [34]. However, given that 94.8% of the 39 cases identified had received treatment, 51.5% of cases with anorexia nervosa had improved, along with two thirds of the cases with bulimia nervosa remitting in their symptomatology, our study cautions against therapeutic nihilism in treating eating disorders in the elderly. Nevertheless, the prognosis of eating disorders in older people is difficult to accurately predict due to the limited nature and number of studies, and time limited follow up in many cases. In addition, it was found that chronicity of illness in older people with anorexia nervosa was associated with either relapse or death as a result of their complications, with illness duration being far longer than that seen in adolescent and younger adults.

There was a lack of consistency in the treatment of eating disorders, even within the sub-diagnoses. This may reflect the changes that have come about in both clinical recognition and the emergence of different treatment modalities, as seen with Ronch’s case study in 1985 [23], where a 75-year-old man had symptomatology strongly suggestive of anorexia nervosa, but due to his age, there appeared to be a hesitation to diagnose him as such; however, the multimodal treatment was successful with resultant weight gain and alteration of food-related behavioural patterns.

Furthermore, the best treatment outcomes were seen in those who were treated through a multidimensional approach, where patients received both therapy and medications (the types of psychotherapy and medication varied significantly between cases) in addition to nutritional support. The current Royal Australian New Zealand College of Psychiatrists clinical guidelines for eating disorder (2014) [56] recommend a multimodal approach, with psychotherapy either family or individual based, along with providing medical and psychiatric indicators and parameters for hospitalisation admissions. Whilst a cautious note was made about limited evidence for psychotropic medications, low-dose olanzapine was recommended; yet it should be taken into account that olanzapine has only recently been employed for treatment of anorexia nervosa [56]; consequently, it is unlikely to have been trialled in early case studies or reports. Due to the greater degree of vulnerabilities to both side effects of medications, as well as medical complications secondary to eating disorders, these guidelines may not be directly translated to elderly patients in the same way as their younger counterparts.

With regards to our research, in almost all of the identified studies, there was no data to suggest that considerations had been made to support older-age–specific factors when designing treatment approaches for eating disorders. This again exemplifies the limited understanding and recognition of eating disorders, which makes it difficult to then tailor treatment approaches for older people with this diagnosis.

### Limitations

There are several limitations relating to internal validity of the studies, publication bias, and variations in diagnostic criteria over time.

#### Internal validity

Our findings regarding treatment are limited by several issues affecting internal validity. These include Rosenthal’s expectancy of advantage, the Hawthorne effect of scrutiny, confounding factors affecting outcomes, and the Placebo effect due to lack of controlled study designs. Expectations of clinicians and patients may have had a positive or negative effect on treatment outcomes. Treatment choice may be closely linked to clinician expectations. Patients, particularly inpatients, are likely to have had some awareness of their being subject to observation, care, and possibly publication of their cases. This could lead to altered outcomes of treatment applied. Consistent data on patient awareness of any of the above was not available. The lack of controlled studies does not allow for an understanding of the placebo effect in this data. Each of these limitations suggests a degree of caution is required in interpreting results relating to treatment efficacy and prognosis.

#### Publication bias

As the issue of new-onset eating disorders in the older-age patient has not been substantially researched, it may also attract a publication bias. In situations of emerging interest such as this, the publication of small, isolated case series or case studies is not unexpected. Interestingly, however, we noted a long publication timeline in our review, which yielded a steady but relatively small number of articles and case reports, 33 and 39 respectively, over a 47-year period. Of these cases, 56% were reported as onset after age 40 years; however, a substantial minority were not claiming a “new onset” case. This is against a backdrop of a relatively large number of publications on eating disorders which focus on adolescents and younger adults and exclude those over 65 years. Notably, only 5 of our 39 cases were published before 1988. The increasing publication of case reports from 1988 onwards may have been stimulated by growth of the discipline of Old Age Psychiatry and increasing interest in clinical observation of this specific group. Whilst this may represent a publication bias, it may also be due to a growing awareness of the need to focus on older people in the domain of eating disorders, due to emerging clinical experience.

#### Variations in diagnostic criteria over time

Although 56% of cases were reported as age of onset after 40 years, this result would be limited by the retrospective nature of data gathering, including recall bias and changing diagnostic criteria for eating disorders over nearly five decades. In the remaining 44% of cases, the stated age of onset was not clearly specified. Interestingly, the preponderance of diagnoses of anorexia nervosa in our review may be related to a longer history of use of this diagnostic category, which was present in DSM I, whilst bulimia and eating disorder not otherwise specified were not added until the 1980s in DSM III. Additionally, there may also be an inherent limitation in using criteria for eating disorders derived from studies of younger adults and adolescents. This may lead to underdiagnosis; however, as specific diagnostic criteria for eating disorders in older patients have not yet been proposed, this is unable to be addressed at present. The evidence derived from case studies and case series is not sufficiently robust to allow for clear description of cases as new onset or late onset. The continuum of symptoms over a life span is not guaranteed, and it is possible that the cases identified by our review have had periods of remission of symptoms, or prior symptoms were not detected or not presented for treatment. We also raise the possibility of a significant limitation in diagnostic accuracy when presenting case series data of anorexia nervosa in older patients. The lack of well described and consistent diagnostic criteria makes it difficult to determine whether published cases represent the disorder of anorexia nervosa or a symptom of anorexia which may be commonly associated with medical conditions in older patients or psychiatric illness such as depression. The absence of clear evidence for accurate diagnosis demonstrated by our review, suggests the need for further treatment studies which utilise standardised diagnostic criteria. It was not the original intention of our review to focus on treatment of new onset eating disorders in the older patient and, whilst our review highlights the possibility of a “late onset” or “new onset” group in the older patient, this label requires substantiation in future studies.

## Conclusions

This is the first study that has systematically reviewed the literature on the treatment of eating disorders in the older population. Our review describes the existing, albeit small, body of evidence for treatment of all identifiable eating disorders in the older patient. The quality of this evidence allows tentative conclusions and suggests a greater body of evidence is needed to support clear directions on treatment of eating disorders in older patients. The review has highlighted that anorexia nervosa was the most commonly diagnosed eating disorder amongst older people. Whilst multimodal approaches to treatment provided the best outcomes, there was no consistent approach to management. Future publications need to provide more comprehensive and consistent descriptions of the clinical cases presented, as well as treatment approaches. Future research should focus on designing and conducting trials which yield age-appropriate clinical treatment guidelines for patients over 65 years of age.

## Data Availability

Not applicable.
